# Ultrasound Guided Hydrodissection of Median Nerve with the Use of 5% Dextrose in Carpal Tunnel Syndrome: A Case Report

**DOI:** 10.31729/jnma.8751

**Published:** 2024-09-30

**Authors:** Sangam Pokharel, Shirish Silwal, Prakash Shrestha, Kabin Neupane

**Affiliations:** 1 Dev Hospital Private Limited, Hospital Road, Narayangadh, Chitwan, Nepal

**Keywords:** *carpal tunnel syndrome*, *dextrose*, *hydrodissection*

## Abstract

Carpal tunnel syndrome is an entrapment neuropathy of median nerve in wrist presenting with symptoms like pain, numbness, and paresthesia in hand and lateral three fingers. Conservative treatments are effective for mild to moderate cases, whereas severe or refractory carpal tunnel syndrome may require surgical intervention. Hydrodissection, a technique involving the separation of the nerve from surrounding tissues, has emerged as a minimally invasive and effective treatment option for carpal tunnel syndrome, leading to faster recovery and lower complication.

In this case report, a 48 years female with two years history of severe pain and paresthesia in right hand, underwent ultrasound-guided hydrodissection of median nerve with 5% dextrose. Following intervention, the patient reported decreased symptoms and expressed satisfaction with the treatment during subsequent follow-up after 2 weeks. Ultrasound-guided hydrodissection of median nerve with 5% dextrose is effective treatment modality for patients with failed conservative management of carpal tunnel syndrome.

## INTRODUCTION

Carpal tunnel syndrome is a common entrapment neuropathy of the median nerve causing paraesthesia, pain, numbness in the distribution of median nerve due to its compression at the carpal tunnel. Conservative measures are often effective in managing mild to moderate cases, severe or refractory cases may require surgical intervention.^1^ Hydro-dissection is an injection technique that separates nerve from the surrounding tissue whereas use of 5% dextrose relieves pain by stimulating anti-inflammatory response through inhibition of Transient receptor potential vanilloid1(TRPV1) capsaicin. Thus, hydrodissection is safe and effective alternative to corticosteroid injections and surgery with early recovery and prolonged relief.^2^ Hereby we report a case of a 48 years female who underwent hydro-dissection for her carpel tunnel syndrome.

## CASE REPORT

A 48 years female presented to the outpatient department with two year history of pain, numbness paresthesia, tingling sensation in right hand. She described the pain as severe which had begun at thenar eminence to lateral three fingers progressing to wrist. She denied any trauma and was systemically well. She had history of hypothyroidism for ten years under levothyroxine 75 microgram per day. There is no other significant medical and surgical history. She is housewife and nulliparous woman. She admits undergoing multiple conservative treatments with drugs as paracetamol, nonsteroidal anti-inflammatory drugs, and pregabalin for more than 6 weeks but was reluctant to surgical exploration as advised previously at other centres.

**Figure 1:**
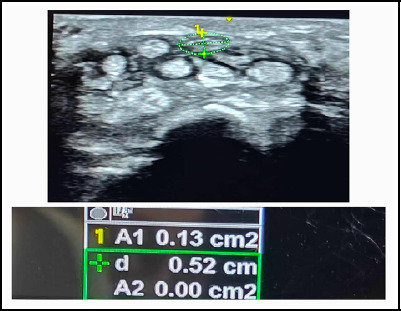
Measurement of cross sectional area of median nerve.

Upon examination vitals were normal. On local examination, no erythema, normal skin temperature noted. No tenderness on superficial and deep palpation. Motor examination was normal. Tinel's sign and Phalens test and Durkan test were positive. Routine laboratory blood tests, thyroid stimulating hormone (5.2 mU/L) and X-rays of the wrist and hand were unremarkable. Nerve Conduction Velocity (NCV) showed positive for compression of median nerve. Ultrasonography showed swollen hypoechoic structure of nerve, with cross sectional area of median nerve at proximal carpal tunnel at the level of scaphoid and pisiform was found to be 13 mm^2^ (Figure 1). It was calculated by ellipse formula (nab/4) and cross sectional area greater than 10 mm^2^ was confirmative of median nerve compression.

The patient was counselled about the intervention, well-written informed consent for the procedure and publication was taken. The patient position was maintained with forearm in supination with slight wrist dorsiflexion. With standard aseptic precautions, high-frequency linear probe was placed 4cm proximal to wrist where median nerve was visualized in transverse plane of wrist. With local infiltration on skin, with use of 27g needle, total 10 ml of 5 % dextrose was prepared and injected via in-plane technique through ulnar approach, 5 ml just below the inferior surface of median nerve and 5 ml above superior surface separating from flexor retinaculum which was then followed by tenotomy that is multiple needle puncture of flexor retinaculum. The patient then monitored for 30 minutes. No adverse events noted then was advised to continue previously taken pregabalin for few weeks in tapering dose as to avoid any unpleasant withdrawal symptoms. During follow up, patient had satisfactory result with symptoms decreased to 50% in response to the treatment as assessed by Numeric Rating Scale.

## DISCUSSION

Hydrodissection of the median nerve is newer technique used for the management of pain due to CTS. The principal cause of nerve injury is due to the gliding resistance of the median nerve within the carpal tunnel and hydrodissection relieves this pressure and thus helps to repair the nerve.^3^ Use of 5% dextrose acts by stimulating an ant-inflammatory response through inhibition of Transient receptor potential vanilloid 1 (TRPV1) capsaicin, causing sensitive receptors to prevent release of substance P and calcitonin gene related peptide.^4^ The strength of our study was ultrasonography guided hydrodissection with dextrose solution which has minimal adverse events with prolonged relief in comparison with steroid use, as described by Mathieu T et al.^2^ This case report focus on single case, so it limits the generalizability of findings and also include short term follow up limiting insights of long term efficacy of treatment.

A prospective randomized controlled trial by Wu et al, shows hydrodissection by 5% dextrose significantly reduces pain and disability, improvement on electrophysiological response measures, and decreased cross-sectional area of the median nerve with effectiveness lasting for about 6 months.^5^ A retrospective study has shown clinically important and durable benefit in those experiencing persistent or recurrent CTS after surgery.^6^

Studies have shown injecting 5% dextrose separate the median nerve from surrounding tissues, is safe and effective treatment for carpal tunnel syndrome and typically is quick and does not require any specialized equipment, making it cost-effective and convenient option for patients and also when compared to corticosteroid injections, normal saline and splint, it has been found to be more effective.^7,8^ Retrospective follow up study has shown, use of hydrodissection with 5% dextrose provides a long term positive outcome in patients with CTS.^9^

Further studies are needed to explore the optimal injection volume, number of injections, and long-term outcomes of ultrasound-guided hydrodissection for CTS.
